# Communications

**Published:** 1891-11

**Authors:** 


### COMMUNICATIONS.

*Editor Journal of Comparative Medicine and Veterinary Archives.*

Dear Sir:

Will you kindly make the following corrections in the report of the proceedings of the United States Veterinary Medical Association, so far as the part which I took in the discussions is concerned, as published in the October number of your Journal.

On page 507 it should read: "*I do not know about Dr. Law, not having seen him lately*," instead of "*I do not know anything about Dr. Law, never having seen him*," as printed. In the seventh line of the same paragraph substitute *would* for *should* and in the eighth line insert the word *have* before *felt;* also in the second line of the last sentence in the same paragraph substitute *may* for *would* before *have*, and *would* for *should* before *like.* In the third line of the last sentence substitute *would* for *should* before *not.* On page 548 in the sixth line from the foot of the page substitute *I have had the* for *it has been my* before *opportunity.* On page 549 in the last sentence but one of the second paragraph place a *period* after cultivation. Substitute *cultures from these colonies* for *and* before *inoculation*, and *inoculated* for inoculation before *into.* Substitute *though* for *if* after even. Insert *original* between *the* and *sick.* Insert *killed the animal* between *animal* and *and.* Insert *were* before *absent* and omit the *last three words* of the sentence. In the last sentence of this paragraph omit the word *other* before *matters* and add to the sentence the words *while Dr. Salmon is right in certain other matters.*

Yours truly,

A. W. CLEMENT.

Baltimore, Md., October 9th, 1891.

---

Boston, Mass., Oct. 14th, 1891.

*Editors Journal of Comparative Medicine and Veterinary Archives.*

Dear Mr. Editor :

I have just received the JOURNAL OF COMPARATIVE MEDICINE AND VETERINARY ARCHIVES for October 1891, containing my article on "Cattle Transportation," read before the United States Veterinary Association at Washington. In our hurry at the meeting and the imperfect condition of my manuscript, I find several errors and omissions, which I beg of you to correct. On page 500 read for "scale sheep"—"sheep scab," and on page 501 the words "poor creatures" ought to come after "stiffs" instead of after the word "situation"—"poor creatures" applied to the "stiffs" not to the agents.

In the discussion that followed, owing to my own deafness and the poor acoustic properties of the hall, which the stenographer complained of also, *I find my remarks entirely unintelligible,* and the remarks of the other speakers quite different from what I understood them to make ; indeed their remarks as printed are so irrelevant that I will defer any criticism of what they are credited with saying until they have time to correct or endorse them.

The remarks attributed to me on page 583 should read :— "The changes enforced at present under the new Regulations for cattle shipments abroad will bring no better results so far as freedom from hardships is concerned than was obtained under the old system when no such regulations had to be enforced in the shipment of animals. The fact is you have attacked the wrong end.

Inland transportation and the class of animals has most to do with the condition in which the cattle are received aboard ship and determine more than any thing else the success of the ocean voyage. You are embarrassing the ships ; I say that is wrong and that somebody is to blame for doing an inconsistent thing, an imperfect piece of work."

Again on page 584 I said :—

"Instead of changing the spaces on the ship it would be better to regulate the size and weight of the animals. Do you mean to tell me that great heavy "distillers" are as good to ship as "stockers," the chances for their going wrong in the stock yard, or any where else being entirely removed? I say further that instead of changing the ships now, you ought to wait and see what grade or class of animals will be allowed to be shipped (should the restrictions abroad be removed). That is the point to be attained. Make this a respectable business, and ship no cattle but those grades and sizes known to be best able to stand the voyage."

In my remarks page 585 with reference to barren mares, I wished to be understood as saying that animals with defects in such important parts were not safe to breed from, many of these changes being the results of domestication and restraint ; they might possibly be perpetuated by animals so affected.

Yours very respectfully.

WILLIAMSON BRYDEN.

---

*Editors Journal of Comparative Medicine and Veterinary Archives.*

Gentlemen :

In your Journal, Vol. XI., 1890, p. 542, is the following from the Chief of the Bureau of Animal Industry:

"I am prepared to say that Texas Fever is not a bacterial disease. There are no peculiar bacteria to be found in the blood ; spleen, liver, or other organs. All the illustrations which may have been published showing preparations of blood from Texas Fever animals swarming with bacteria, and sections of tissues showing the same micro-organisms are misleading and of no value to the student of the disease."

It would be either a bold man, sure of his ground, or one, who was reckless of consequences, who would make so positive an assertion as the above in the face of existing facts.

In contradiction thereto, I now say (and am willing to suffer the consequences,) that a germ is the cause of Texas Fever, and that it is the same germ previously described by me, and found in four different outbreaks of Texas Fever in Nebraska cattle, the original specimens from which can be seen at any time in this laboratory.

I also pronounce the assertion of the Bureau of Animal Industry that Texas Fever is due to a zoospore or some form of malarial parasite to be wrong.

Recently I received from Dr. Tait Butler, V. S., Agricultural College, Miss., a box of ticks. Selecting one of the best and plumpest, I washed it in corrosive sublimate, then in alcohol, then in distilled water and cut open its neck with a sterilized knife. From the cut oozed out a thick, black semi-fluid material. In the same smear proportions gave a germ looking like the one previously described by me; cultures from this material were pure, as proven by tests, and all other tests gave the same germ. Inoculation in cattle produced unequivocal Texas Fever, and from the dead cattle absolutely pure cultures were obtained, and blood specimens give the same germ. Nothing can be plainer, but I have taken the precaution to have trustworthy officials of the University see every step I have made. Cultures have been sent to Prof. Welch, of Johns Hopkins, and to Koch's Laboratory at Berlin, requesting that the experiments be repeated.

Respectfully yours,

F.S. BILLINGS, *Director*,
*Patho-Biological Laboratory of the*
*State University*, *Lincoln*, *Neb.*

